# Genome size expansion and the relationship between nuclear DNA content and spore size in the *Asplenium monanthes* fern complex (Aspleniaceae)

**DOI:** 10.1186/1471-2229-13-219

**Published:** 2013-12-20

**Authors:** Robert J Dyer, Jaume Pellicer, Vincent Savolainen, Ilia J Leitch, Harald Schneider

**Affiliations:** 1 Department of Botany, Natural History Museum, London SW7 5BD, UK; 2 Imperial College London, Silwood Park Campus, Ascot, Berkshire SL5 7PY, UK; 3 Centre for Ecology and Hydrology, Wallingford, Oxfordshire OX10 8BB, UK; 4 Jodrell Laboratory, Royal Botanic Gardens, Kew, Richmond, Surrey TW9 3AB, UK; 5 State Key Laboratory of Systematic and Evolutionary Botany, Institute of Botany, The Chinese Academy of Sciences, Beijing, China

**Keywords:** Genome size, Chromosome size, DNA content, C-value, Monoploid, Holoploid, Polyploidy, *Asplenium monanthes*, Apomixis, Spore size

## Abstract

**Background:**

Homosporous ferns are distinctive amongst the land plant lineages for their high chromosome numbers and enigmatic genomes. Genome size measurements are an under exploited tool in homosporous ferns and show great potential to provide an overview of the mechanisms that define genome evolution in these ferns. The aim of this study is to investigate the evolution of genome size and the relationship between genome size and spore size within the apomictic *Asplenium monanthes* fern complex and related lineages.

**Results:**

Comparative analyses to test for a relationship between spore size and genome size show that they are not correlated. The data do however provide evidence for marked genome size variation between species in this group. These results indicate that *Asplenium monanthes* has undergone a two-fold expansion in genome size.

**Conclusions:**

Our findings challenge the widely held assumption that spore size can be used to infer ploidy levels within apomictic fern complexes. We argue that the observed genome size variation is likely to have arisen via increases in both chromosome number due to polyploidy and chromosome size due to amplification of repetitive DNA (e.g. transposable elements, especially retrotransposons). However, to date the latter has not been considered to be an important process of genome evolution within homosporous ferns. We infer that genome evolution, at least in some homosporous fern lineages, is a more dynamic process than existing studies would suggest.

## Background

Homosporous ferns are renowned for their high chromosome numbers, e.g. *Ophioglossum reticulatum* has the highest chromosome number (2*n* = ca.1400) so far reported for any eukaryote [1]. Moreover, the mean chromosome number for homosporous ferns (*n* = 57.05) is far higher than any other plant group (including heterosporous ferns, *n* = 13.6; and angiosperms, *n* = 15.99) [2,3]. The reason for this disparity between land plant groups remains enigmatic and is a major focus of ongoing research in this field (see [3-5]).

The application of novel genome-wide analytical methods, including genome size analysis, is providing significant insight into the processes that shape homosporous fern genomes [3,4,6]. Although our knowledge of genome sizes in ferns is limited (< 1% of species have been analysed), available data suggest that patterns of genome size evolution, which include (i) polyploidisation; (ii) paleopolyploidsation; and, (iii) changes in chromosome size, are not operating uniformly across all fern lineages [6-10].

In contrast to the extreme diversity of genome sizes encountered in angiosperms, which range c. 2,400-fold [11], genome sizes in ferns (monilophytes) are less variable, ranging just c. 94-fold [8,9]. In fact, if we focus within homosporous ferns, this variation in nuclear DNA content only spans c. 25-fold, from 1C = 2.95 pg in *Athyrium filix-femina*[12] to 1C = 72.68 pg in *Psilotum nudum* var. *rubra*[10]. While some of this diversity arises from polyploidy (e.g. *Ophioglossum petiolatum*, 2n = 32*x* = c. 960, and 1C = 65.55 pg, see [10]), genome size changes can also arise within the same ploidy level in some genera. For example, the 1.5-fold range of genome sizes encountered in *Davallia* have taken place at the diploid level (2*n* = 80) with the different genome sizes between species reflected in contrasting chromosome sizes [10]. Such diversity may be attributed to arise through different balances between the amplification of repetitive DNA such as transposable elements (TE) (especially retrotransposons) leading to genome and chromosome size increases and DNA elimination, as frequently observed in angiosperms [13,14].

Available cytogenetic data indicates that apart from a few examples [15], most homosporous ferns are characterized by possessing small and rather conserved chromosome sizes with little evidence of retrotransposon activity [3,6,16,17]. These studies would indicate that genome size variation in homosporous ferns is largely driven by polyploidy. This hypothesis is supported by the relatively small variation (compared with angiosperms) reported in the monoploid genome size (1C*x-*value; 1C*x* = 2.95 pg - 21.02 pg) and the correlation between 2C-value (DNA content of the whole chromosome complement) and chromosome numbers [6]. The monoploid genome size (1C*x*-value) is the DNA content of one un-replicated chromosome set, whilst the holoploid genome size (1C-value) is the DNA content per haploid chromosome complement (see [18]).

In ferns, studies on closely related species in *Dryopteris*[19,20] and *Polypodium*[21] have shown that genome size can be a powerful marker for taxonomic delimitations. However, as far as we are aware, no study to date has investigated the evolution of genome size in a closely related group of fern species, or determined whether it is correlated with breeding system or any morphological traits. Studies in angiosperms have demonstrated that genome size is correlated with several ecological and morphological traits such as seed mass and stomatal density (e.g. [22-25]). Similar studies would be highly informative in ferns, as traits such as spore size and stomatal cell size are often used to infer changes in ploidy levels among closely related species [26-28]. This is based on the implicit assumption that species with higher ploidy levels (and hence larger genomes) will have larger spores, although this has never been systematically tested for genome size within a phylogenetically well defined group.

**Figure 1:**
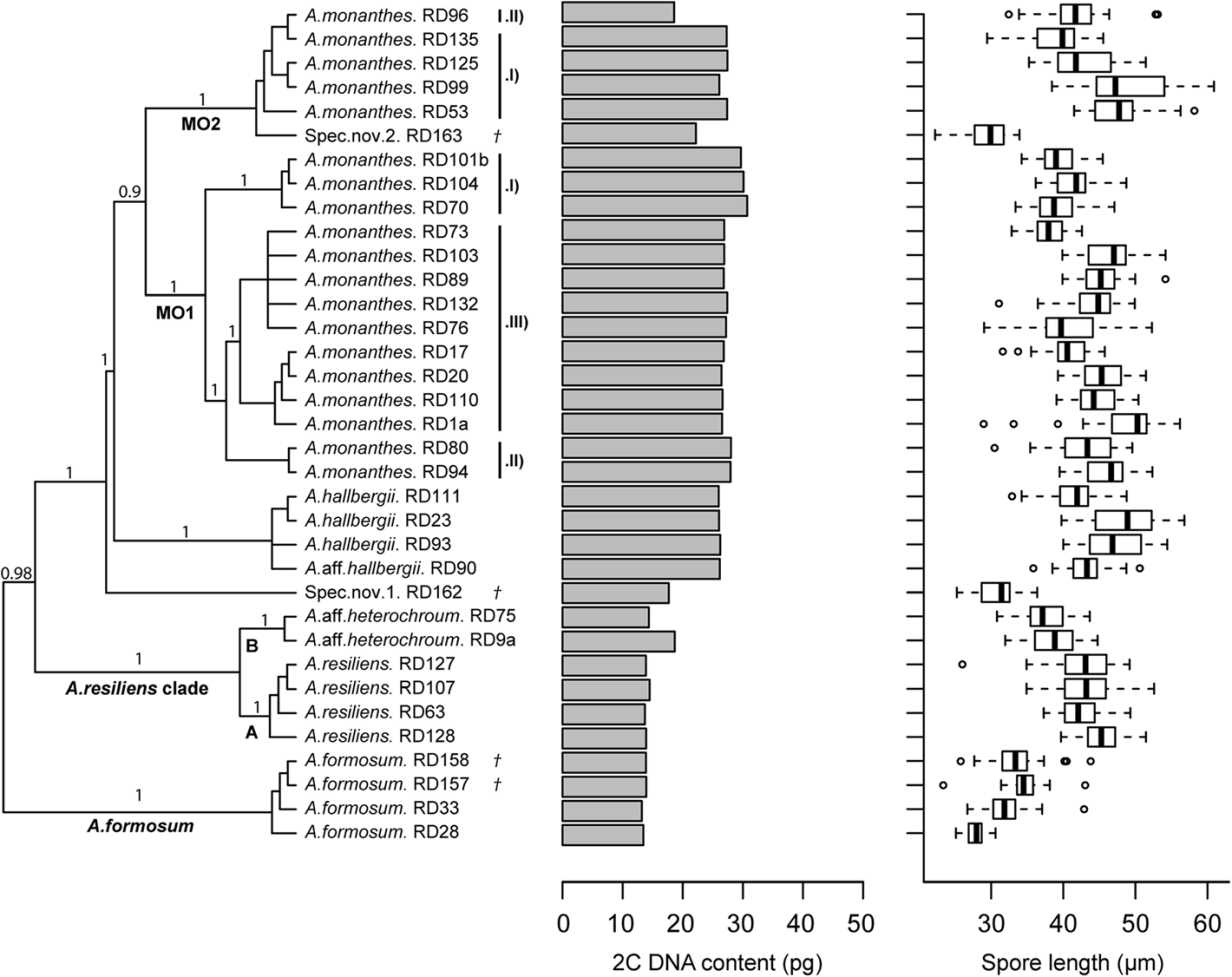
**Phylogenetic framework (based on Bayesian analysis) of the plastid genome, as presented in Dyer *et al*. **[29]**, of the *Asplenium monanthes *complex and related lineages, together with nuclear DNA content and spore length for each specimen analysed.** Tree is rooted according to [29]. Posterior branch support (≥0.8) is shown. 2C DNA content for each specimen is represented by a bar chart, whilst spore length data are shown as box plots. Each boxplot represents the variance of measurements of spores within each specimen, the thick horizontal line is the median, the box indicates the variation observed between the 25th and 75th percentiles, the whiskers show the variance range, and small circles identify extreme outliers. MO1 and MO2 indicate the two distinct *A.monanthes* clades and these are further divided into sub-clades I and II, and also III in the case of MO1 only. *†* symbols indicate samples for which 2C DNA content measurements were made from 6 month old silica material.

In this study we investigate the evolution of genome size and spore size within the *Asplenium monanthes* complex (Figure 1). The complex includes a group of closely related species whose phylogenetic relationships have been recovered, showing evidence of reticulate evolution and multiple apomictic lineages [29]. In addition, polyploidy is known to occur based on previously reported chromosome counts in some taxa (see Methods and [29]).

Our work had three aims: First, we investigated the validity of using C-value measurements obtained from silica dried material. Here, we report the extent of differences in C-value estimates obtained from measurements of fresh and silica dried material (stored for 6 months and/or 2 years). Second, we tested the widely held assumption that DNA amount and spore size are correlated. For this, we conducted comparative analyses between DNA content and spore length for multiple taxa within the *A.monanthes* complex. Third, we examined genome size evolution and determined to what extent genome size variation reflected changes in chromosome size or ploidy levels within this group of ferns. To do this, genome size estimations were made for taxa with known ploidy levels (i.e. karyologically determined in previous studies) to infer DNA ploidies for those species without reported chromosome counts. The data were then analysed within the phylogenetic framework of Dyer *et al*. [29] to provide insights into genome size dynamics.

## Results

### Shifts in genome size associated with the preservation of plant material

Since fresh material was not available for all the specimens studied, the impact of silica drying on the relative fluorescence of nuclei and hence genome size estimates was investigated. Genome size estimates were very similar between fresh leaf material and material that had been stored in silica for 6 months. This was shown for *A.formosum* where genome size estimates for RD28 and RD33 (2C = 13.46 pg and 13.19 pg respectively) were very similar to those obtained from the 6 month old silica dried samples of *A.formosum* RD28, RD33, RD157 and RD158 (2C = 13.95 pg, 13.82 pg, 13.93 pg and 13.87 pg respectively) (Table 1). We therefore inferred no meaningful shift in the genome size measurements for specimens stored in silica for six months and thus genome size estimates obtained from four 6 month old silica samples (*A.formosum*, RD157 and RD158; spec.nov.1, RD162; and spec.nov.2, RD163), were used in all analyses as no fresh material was available.

**Table 1:** Observed shifts in 2C DNA content shown in response to drying and storage of leaf material in silica

		**Fresh material**		**Silica dried material**			
** Species**	**Voucher**	**2C-value (pg)**	**CV%**	**2C-value (pg)**	**CV%**	**Silica age**	**Percentage increase (%)**
*A.formosum*	RD28	13.46 ± 0.03	3.77	13.95 ± 0.04	2.45	6 months	3.55
				17.23 ± 0.12	3.41	2 years	21.88
*A.formosum*	RD33	13.19 ± 0.03	2.73	13.82 ± 0.04	3.87	6 months	4.55
				18.87 ± 0.19	6.74	2 years	30.09
*A.formosum*	RD157	(13.19-13.46)*	-	13.93 ± 0.23	3.15	6 months	(3.37-5.31)
*A.formosum*	RD158	(13.19-13.46)*	-	13.87 ± 0.11	2.24	6 months	(2.95-4.90)
*A.monanthes*	RD70	30.72 ± 0.78	3.94	33.33 ± 0.10	5.98	2 years	7.83
*A.monanthes*	RD94	27.96 ± 0.19	3.36	33.44 ± 0.14	6.75	2 years	16.4
*A.resiliens*	RD107	14.50 ± 0.04	3.07	17.43 ± 0.17	5.39	2 years	16.81
Spec.nov.1	RD162	-	-	17.67 ± 0.19	4.26	6 months	-
Spec.nov.2	RD163	-	-	22.20 ± 0.28	3.54	6 months	-

CV% indicates the coefficient of variation of the 2C DNA peak in the flow histogram. Percentage increase indicates the percentage difference between fresh 2C DNA content and silica-dried 2C DNA content.

*Range of 2C-values obtained from the analysis of fresh leaf material of the same species but different accessions.

In contrast, samples processed using 2 year old silica material showed that 2C-values were on average, 18.6% higher (ranging from 7.83–30.09%), compared with estimates made from fresh material (Table 1). Furthermore, the quality of the measurements made on 2 year old silica samples was notably lower compared with both fresh samples and samples 6 months in age, based on the mean CV% of the peaks in the flow histograms (i.e. fresh samples = 3.37; silica samples 6 month old = 3.25; Silica samples 2 years old = 5.65). Data from 2 year old material were therefore excluded from further analysis.

### Variation in genome size and spore size

Within the *A.formosum* and *A.resiliens* clades, 2C-values were highly conserved (13.19-13.93 pg and 13.70-14.50 pg respectively), as were the spore lengths (27.85-34.56 μm and 42.26-45.07 μm respectively) (Figure 1). All accessions of *A.resiliens* formed a well supported monophyletic lineage (clade A, Figure 1), with accessions of *A.*aff.*heterochroum* occupying a sister position (clade B, Figure 1). Of the two specimens investigated of *A.*aff*.heterochroum*, one (RD75, 2C = 14.38 pg) had a similar 2C DNA content to *A.resiliens* (mean 2C of four individuals = 13.99 pg), while the other was distinctly higher (RD9a, 2C = 18.67 pg). In contrast to this genome size variability, both specimens showed very similar spore lengths (37.43-38.62 μm), which were smaller compared with the mean value for the four *A.resiliens* specimens analysed (43.29 μm)*.*

Within the *A.monanthes* clade, the smallest genome size was found in the diploid spec.nov.1, which has been shown to be sister to the rest of the clade (Figure 1). Diploid sexual, spec.nov.2 showed a distinctly larger genome size (22.20 pg). The specimens of *A.hallbergii* and *A.*aff.*hallbergii*, which form a sister clade to the main clades of *A.monanthes* (MO1 and MO2), showed very little variation in nuclear DNA contents (2C = 26.00-26.23 pg), but a relatively large variation in spore length (41.33-48.69 μm). Within the *A.monanthes* MO1 lineage, three sub-clades with similar, but distinct 2C value ranges could be identified: MO1 (I), 29.68-30.72 pg; MO1 (II), 27.96-28.03 pg; and MO1 (III), 26.45-27.41 pg. Spore size across these three sub-clades varied from 38.09 μm to 48.54 μm. The nuclear DNA content of individuals within the *A.monanthes* (MO2) clade was found to be similar (2C = 26.09-27.43 pg), with the exception of a single specimen (RD96, designated MO2 II), which had a significantly smaller genome size (2C = 18.58 pg) (Figure 1 and Table 2). Spore size varied from 38.94-48.54 μm, and the value for specimen RD96 also fell within this range (42.11 μm).

**Table 2:** A summary of the specimens analysed including, mean 2C DNA content and mean spore length measurements per specimen (both with standard deviation values)

**Species**	**Voucher**	**Mean spore length (μm)**	**Mean 2C-value (pg)**	**CV%**	**Reproductive mode**
A.fibrillosum*	RD10b	47.90 ± 8.14	37.78 ± 0.18	6.95	Sexual
*A.formosum*	RD28	27.85 ± 1.26	13.46 ± 0.03	3.77	Sexual
*A.formosum*	RD33	32.20 ± 3.80	13.19 ± 0.03	2.73	Sexual
*A.formosum†*	RD157 (IJ2436)	34.65 ± 3.37	13.93 ± 0.23	3.15	Sexual
*A.formosum†*	RD158 (ES1398)	33.56 ± 3.78	13.87 ± 0.11	2.24	Sexual
*A.hallbergii*	RD23	48.69 ± 5.18	26.05 ± 0.06	3.72	Apomictic
*A.*aff*.hallbergii*	RD90	43.32 ± 3.10	26.17 ± 0.12	3.47	Apomictic
*A.hallbergii*	RD93 (RD112)	47.63 ± 4.81	26.23 ± 0.19	3.40	Apomictic
*A.hallbergii*	RD111	41.33 ± 3.91	26.00 ± 0.06	3.24	Apomictic
*A.*aff*.heterochroum*	RD9a	38.62 ± 3.35	18.67 ± 0.08	3.11	Apomictic
*A.*aff*.heterochroum*	RD75	37.43 ± 3.19	14.38 ± 0.05	2.39	Apomictic
*A.monanthes* (MO1) I	RD70	39.03 ± 3.35	30.72 ± 0.78	3.94	Apomictic
*A.monanthes* (MO1) I	RD101b	39.44 ± 2.96	29.68 ± 0.12	4.19	Apomictic
*A.monanthes* (MO1) I	RD104	41.85 ± 3.73	30.11 ± 0.32	4.15	Apomictic
*A.monanthes* (MO1) II	RD80	43.15 ± 4.53	28.03 ± 0.06	2.75	Apomictic
*A.monanthes* (MO1) II	RD94	45.79 ± 3.31	27.96 ± 0.19	3.36	Apomictic
*A.monanthes* (MO1) III	RD1a	48.54 ± 5.94	26.54 ± 0.16	4.05	Apomictic
*A.monanthes* (MO1) III	RD17	40.31 ± 3.34	26.84 ± 0.52	4.35	Apomictic
*A.monanthes* (MO1) III	RD20	45.53 ± 3.38	26.45 ± 0.39	3.77	Apomictic
*A.monanthes* (MO1) III	RD73	38.09 ± 2.42	26.91 ± 0.14	4.08	Apomictic
*A.monanthes* (MO1) III	RD76	40.78 ± 5.07	27.21 ± 0.13	3.82	Apomictic
*A.monanthes* (MO1) III	RD89	45.30 ± 3.24	26.82 ± 0.02	3.14	Apomictic
*A.monanthes* (MO1) III	RD103	46.39 ± 3.54	26.90 ± 0.14	3.64	Apomictic
*A.monanthes* (MO1) III	RD110	44.54 ± 2.85	26.62 ± 0.16	4.04	Apomictic
*A.monanthes* (MO1) III	RD132	43.97 ± 4.27	27.41 ± 0.26	4.03	Apomictic
*A.monanthes* (MO2) I	RD53 (RD45)	47.76 ± 4.27	27.40 ± 0.09	3.53	Apomictic
*A.monanthes* (MO2) I	RD99	48.54 ± 5.88	26.09 ± 0.24	5.29	Apomictic
*A.monanthes* (MO2) I	RD125	42.80 ± 4.36	27.43 ± 0.18	4.39	Apomictic
*A.monanthes* (MO2) I	RD135	38.94 ± 3.69	27.32 ± 0.06	3.92	Apomictic
*A.monanthes* (MO2) II	RD96	42.11 ± 4.82	18.58 ± 0.09	3.00	Apomictic
A.polyphyllum*	RD98 (RD95)	35.42 ± 3.58	29.95 ± 0.10	4.02	Sexual
*A.resiliens*	RD128	45.07 ± 2.96	13.90 ± 0.07	2.75	Apomictic
*A.resiliens*	RD63	42.39 ± 2.93	13.70 ± 0.12	2.80	Apomictic
*A.resiliens*	RD107	43.45 ± 4.35	14.50 ± 0.04	3.07	Apomictic
*A.resiliens*	RD127 (RD64)	42.26 ± 4.59	13.87 ± 0.07	2.85	Apomictic
A.soleirolioides*	RD82 (RD71)	39.37 ± 2.89	50.29 ± 0.16	4.03	Sexual
Spec.nov.1*†*	RD162 (JM1339)	30.87 ± 2.80	17.67 ± 0.19	4.26	Sexual
Spec.nov.2*†*	RD163 (SK10151)	29.71 ± 2.63	22.20 ± 0.28	3.54	Sexual

Voucher numbers are linked to accession numbers for sequences deposited at Genbank [29]. In cases where alternate voucher numbers appear in brackets, these accessions were used for phylogenetic analysis. CV% indicates the mean coefficient of variation of the 2C DNA peak for each specimen. Reproductive mode was determined from spore number per sporangia, and some prothalli observations [29]. MO1 and MO2 refer to the two clades within the *A.monanthes* complex identified by the phylogenetic analysis of Dyer *et al. *[29].

*Data for these species (which belong to the *A. castaneum* clade) were not considered to be reliable as they were estimated from 2 year old silica dried material and were therefore not included in analysis.

*†*Data for these species were estimated from 6 month old silica dried material.

*Asplenium fibrillosum, A.soleirolioides,* and *A.polyphyllum*, which together comprise the *A.castaneum* clade*,* were observed to have the highest 2C values in the complex (37.78 pg for *A.fibrillosum,* 29.95 pg for *A.polyphyllum*, and 50.29 pg for *A.soleirolioides*) (Table 2 and Table 1), but since these estimates were made from material that had been stored on silica for 2 years their reliability is questioned (as noted above). Interestingly, the mean spore length of the *A.fibrillosum* specimen was also very large (47.90 μm), although those for *A.soleirolioides* and *A.polyphyllum* were distinctly smaller (39.37 μm and 35.42 μm respectively).

#### Correlation between DNA amount and spore size

**Figure 2:**
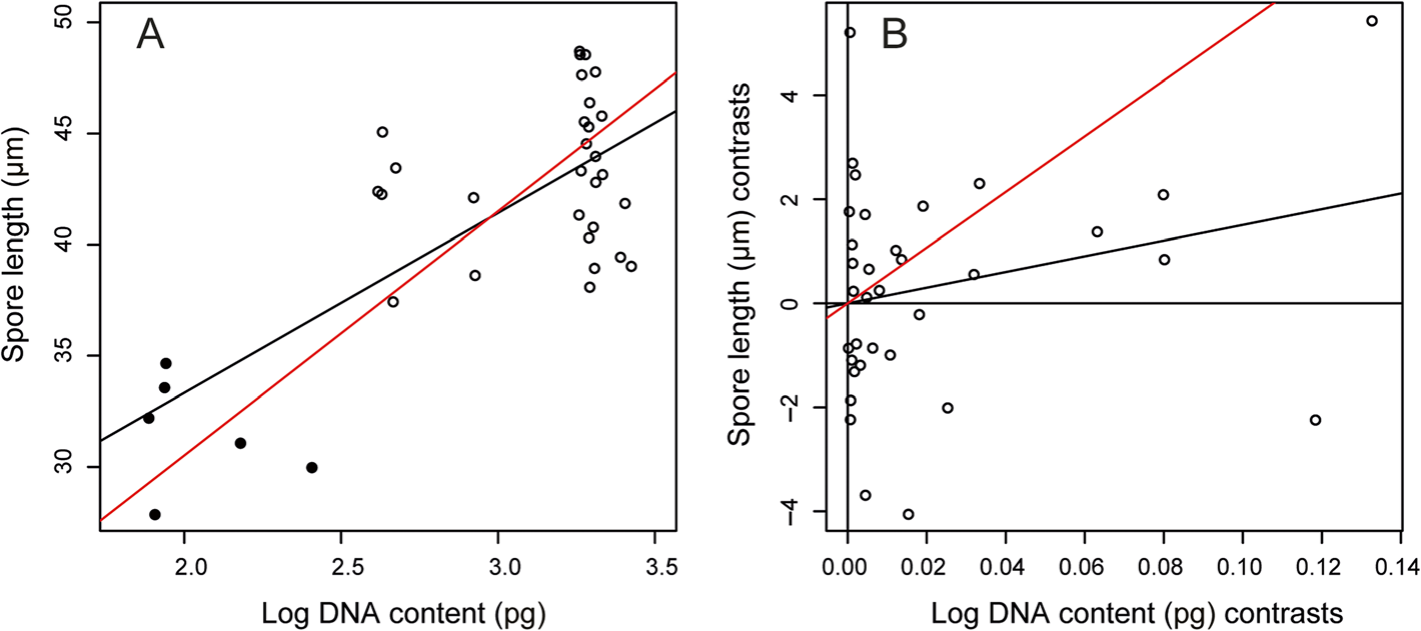
**Regression analyses of 38 specimens showing the positive relationship between DNA amount and spore length for both (A) the raw data (p = 4.57x10**^**-6**^**, *r***^***2***^ **= 0.542, slope = 10.986, 95% CI’s = 8.665-13.929), and (B) data from the phylogenetically independent contrasts analysis (Branch transformation of Grafen; SMA, p = 0.101) (see Table **3**).** DNA amount values were logged prior to analysis, and the values used for DNA amount were based on reproductive mode: i.e. 2C-values were used for apomictic taxa, and 1C-values were used for sexually reproducing taxa. Lines of best fit are indicated using LM (black line) and SMA (red line) models, and in B only, the SMA is forced through the origin. In plot A, open circles represent apomictic taxa, and filled black circles represent sexually reproducing taxa.

Regression analysis of the raw data showed a strong positive correlation (p = 4.57×10^-6^, *r*^*2*^ = 0.542, slope = 10.986, 95% CI’s = 8.665-13.929) between DNA amount and spore length (Figure 2A). However, phylogenetically independent contrasts analysis indicated no significant relationship between contrasts (Grafen branch transformation, p = 0.101) (Figure 2B and Table 3). This indicated that divergences in DNA amount are not associated with divergences in spore length, and therefore there is no correlated evolution between DNA amount and spore length.

**Table 3:** Phylogenetically independent contrasts (PICs) analysis of the relationship between genome size and spore length

**Branch transformation method**	**Phylogenetic signal (κ)**				**PIC standardisation**			
	**GS**		**SL**		**GS**		**SL**	
	***κ***	***P***	***κ***	***P***	***r***^***2***^	***P***	***r***^***2***^	***P***
Log	2.500	**0.001**	0.773	**0.001**	0.314	**0.024**	0.207	0.076
Pagel	2.814	**0.001**	0.800	**0.001**	0.196	**0.008**	0.021	0.408
Nee	2.317	**0.001**	0.988	**0.001**	0.176	**0.014**	0.019	0.434
Grafen	1.131	**0.001**	0.273	**0.003**	0.035	0.289	0.080	0.090

DNA amount values were log transformed prior to analysis. GS = genome size, and SL = spore length. 1C or 2C DNA values were used depending on the reproductive mode: 2C-values are used for apomictic taxa, and 1C-values are used for sexual taxa. Results are given for four branch length transformation methods, as discussed in the Materials and Methods. Significant P values are given in bold.

### Genome size and ploidy level

**Figure 3:**
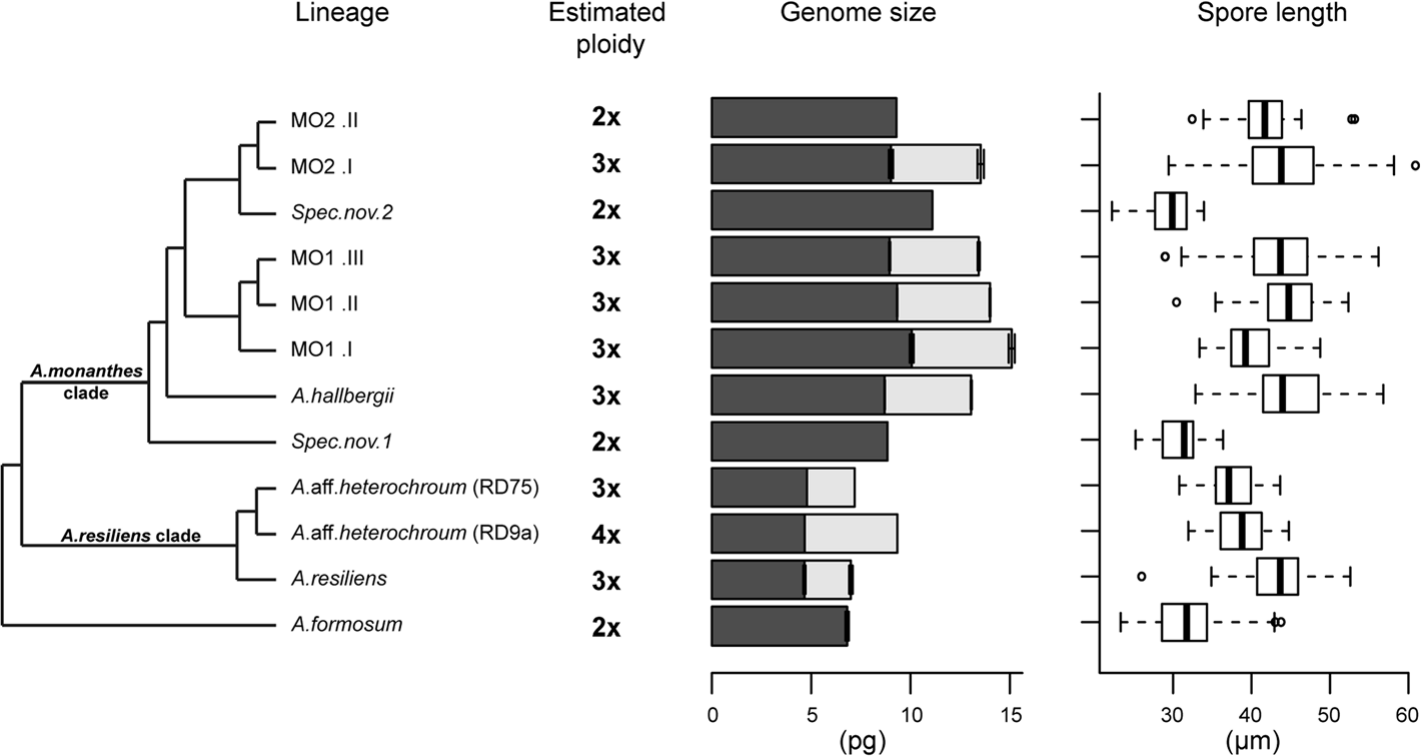
**A summarized phylogenetic tree of the *Asplenium monanthes *complex and related lineages [as presented in 29] together with the inferred ploidy levels (based on published chromosome data), mean holoploid genome size (1C-value; grey bars), and inferred monoploid genome size (1C*x*-value; black bars) and the mean spore length (μm) of each operational unit.** Posterior branch support >0.8 is shown. For diploids only black bars are present as the 1C-value is equal to the 1C*x*-value. Standard error bars are included for 1C-value and 1C*x*-value means, except for lineages represented by one specimen only. Tree is rooted according to Dyer *et al*. [29]. Details of the box plots for spore size are given in the legend to Figure 1.

Monoploid genome size (1C*x*-values) and ploidy levels were not inferred for *A.fibrillosum*, *A.soleirolioides* and *A.polyphyllum*, due to the shifts observed in the 2C DNA contents of these species, as a consequence of their storage for 2 years in silica (see above). Ploidy levels and 1C*x*-values were inferred for all other subclades analysed (Table 4 and Figure 3).

**Table 4:** **A summary of the genome size and spore size data obtained for specimens belonging to the *Asplenium monanthes *clade and related lineages**

**Species**	**Mean 2C-value (pg)**	**Mean CV%**	**Inferred Ploidy (*x*)**	**Holoploid 1C-value (pg)**	**1C-value SE**	**Monoploid 1C *x *-value (pg)**	**1C *x *-value SE**	**Mean spore length**
*A.formosum*	13.61 ± 0.10	2.97	2	6.81	0.09	6.81	0.09	32.07
***A.resiliens *clade**								
*A.resiliens*	13.99 ± 0.08	2.87	3	7.00	0.09	4.66	0.06	43.29
*A.*aff*.heterochroum* RD9a	18.67 ± 0.08	3.11	4	9.34	-	4.67	-	38.62
*A.*aff*.heterochroum* RD75	14.38 ± 0.05	2.39	3	7.19	-	4.79	-	37.43
***A.monanthes *clade**								
*A.hallbergii*	26.11 ± 0.11	3.46	3	13.06	0.03	8.70	0.02	45.24
MO1 I	30.17 ± 0.41	4.09	3	15.09	0.15	10.06	0.10	40.11
MO1 II	28.00 ± 0.13	3.06	3	14.00	0.02	9.33	0.01	44.47
MO1 III	26.86 ± 0.21	3.88	3	13.43	0.05	8.95	0.03	43.72
MO2 I	27.06 ± 0.14	4.28	3	13.53	0.16	9.02	0.11	44.51
MO2 II	18.58 ± 0.09	3.00	2	9.29	-	9.29	-	42.11
Spec.nov.1	17.67 ± 0.19	4.26	**2**	8.84	-	8.84	-	30.87
Spec.nov.2	22.20 ± 0.28	3.54	**2**	11.10	-	11.10	-	29.71

Mean 2C DNA content (pg) and mean spore length (μm) were only calculated for sub-clades distinguished by low genome size variation (Figure 1). This resulted in separate inferences of genome size and ploidy for the two *A*.aff.*heterochroum* specimens (RD75 and RD9a), and *A.monanthes* subclades: MO1 (I); MO1 (II); MO1 (III); MO2 (I), and MO2 (II) (Figure 1). Published chromosome counts (*A.formosum*, 2*n* = 2*x* = 72; *A.monanthes*, 2*n* = 3*x* = 108; *A.resiliens*, 2*n* = 3*x* = 108) were used to estimate ploidy and hence calculate monoploid genome size (i.e. 2C-value divided by ploidy level). For lineages with unknown ploidy levels, monoploid genome size and ploidy level were estimated according related species. Estimates of ploidy underlined in bold are based on the spore number and spore measurements [29].

Within the *A.resiliens* clade, different ploidy levels were inferred for the two *A*.aff.*heterochroum* specimens. One specimen of *A.*aff.*heterochroum* (RD75; 2C = 14.38 pg) had a similar 2C-value to *A.resiliens* (mean 2C = 13.99 pg)*,* a species reported to be triploid, suggesting that RD75 could also be a triploid. In contrast, the other *A*.aff.*heterochroum* specimen analysed (RD9a) had a significantly higher 2C-value (18.67 pg) and a 1C*x*-value approximately four times that inferred for RD75, which lead to the inference that this specimen was a tetraploid.

The suggestion that most *A.monanthes* specimens are triploid is based on the observation that, with the exception of one report of a tetraploid count, all counts for this species have been 2*n* = 3*x* = 108. Under this assumption, the mean 1C*x*-values for MO1 sub-clades I, II and III are 10.06 pg, 9.33 g, and 8.95 pg, respectively (Table 4). A triploid level is also assumed for MO2 (I), based on the 2C-values, resulting in a mean 1C*x*-value of 9.02 pg. The *A.monanthes* MO2 (II) sub-clade, which comprised a single specimen (RD96), had a significantly lower 2C-value of 18.58 pg, and we infer that this specimen is likely to be a diploid cytotype. This would result in a 1C*x* value of 9.29 pg, which is in broad agreement with the monoploid genome sizes calculated for the remaining *A.monanthes* sub-clades (Table 4). Nevertheless, it is noted that the mean spore size of RD96 (42.11 μm) conflicts with this finding, suggesting that this specimen is a polyploid.

In *A.hallbergii*, the similarity of its mean 2C-value (26.11 pg) and mean spore size (45.24 μm) to that of the triploid *A.monanthes* lineages (MO1 and MO2.I) suggests this species is also a triploid. As for the two new species, spec.nov.1 and spec.nov.2, they differ in 2C-values (17.67 pg and 22.20 pg respectively) but have similarly small spore sizes. They are currently inferred to be diploid although further data are needed to confirm or refute this.

Overall, the results show that monoploid genome size varies 2.4-fold (4.66-11.10 pg), with little variation in 1C*x*-values within clades, but considerable variation between some of them (Table 4 and Figure 3). For example, compared with the small 1C*x*-values in the *A.resiliens* clade (4.66-4.79 pg), the mean 1C*x*-value for diploid *A.formosum* (6.81 pg) was markedly higher. A larger monoploid genome size was also noted in the *A.monanthes* clade compared with the *A.resiliens* clade. Indeed, the largest monoploid genome sizes were encountered within the *A.monanthes* clade (8.70-11.10 pg), with spec.nov.2 having the highest 1C*x*-value (11.10 pg) of all, which is more than double the mean monoploid genome size of the *A.resiliens* clade.

## Discussion

### Considerations for the use of silica dried material for genome size estimation

The question as to whether it is possible to use silica preserved material to measure genome size in absolute units has recently become the focus of intense debate [30-32]. In order to avoid ambiguous interpretations, some authors only advocate the use of fresh material to estimate genome size [33], and restrict the use of silica-dried material to estimating DNA ploidy levels (e.g. [34]). However, recent studies have started to explore the use of silica dried material for genome size measurements (see [30]). This would help overcome some of the constraints imposed by the need for fresh material, especially when the quality of measurements is not compromised (i.e. <5% of variation between fresh and silica-dried tissues).

In the current analysis some of the accessions were only available as silica dried material (either 6 months or 2 years old), so the reliability of the genome size estimates obtained from such material was investigated. We were able to compare fresh and silica-dried tissues belonging to the same accession, for three separate species (Table 1). We found a significant increase in fluorescence intensity in all silica-dried samples of 2 years in age, relative to the fresh material, which resulted in genome size estimates up to c. 30% larger compared with those estimated on fresh material (Table 1). In addition, as previously reported, %CV was also seen to increase, and in most cases these exceeded acceptable values (i.e. >5%). We therefore conclude that for *Asplenium*, the use of 2 year old silica dried material is unsuitable for obtaining accurate genome size estimations.

In contrast, samples of two accessions of *A.formosum* stored for 6 months in silica gave very similar results to those obtained from fresh material (RD28 and RD33; Table 1). In addition, there was no evidence of any reduction in the quality of the flow histogram with the CVs for both fresh and silica samples being similar. Nevertheless, while these results suggest that the short term storage of *Asplenium* samples in silica is suitable for genome size estimations, the extent to which these findings can be extrapolated to other plant genera, including other ferns remains unknown.

### Relationship between DNA amount and spore size

Numerous studies in angiosperms have shown a positive correlation between genome size and a number of morphological traits, including seed mass, cell size and stomatal density (e.g. [23,24,35,36]). Beyond the angiosperms, such studies are relatively rare (e.g. [19,21]), and while spore size is often used as a proxy for inferring ploidy level (e.g. [26-28]), there have been no empirical studies to date that test these inferences using genome size. In this study we investigated the relationship between genome size and spore size to determine the extent to which such an assumption is valid.

We found a significant and positive correlation between nuclear DNA amount and spore length using the raw data. However, when evolutionary relationships were considered, using phylogenetically independent contrasts (PICs), no significant correlation was found. The discrepancy between analyses is interesting, and although the significance of the raw data should not be discounted it does highlight the importance of using PICs to determine the evolutionary association of statistically non-independent traits [37].

The insignificant relationship observed between DNA amount and spore size calls into question the use of spore size for inferences of ploidy level within homosporous ferns. Previous authors have pointed out that this inference should be restricted to very close relatives, and the distinction between diploids and their autoploid offspring [26,27]. Our findings are consistent with these suggestions. In addition, our study sample is mainly comprised of apomictic accessions, and due to a low sample number of sexually reproducing species we are unable to test for the effect of reproductive mode on the relationship between traits.

Considerable variation was found in spore length, both within and between specimens of the apomictic lineages. In part this variation may be a consequence of the non-globulose shape of the monolete spores found in this complex, making precise measurements difficult. However, the variation may also be due to the variable nature of Döpp Manton sporogenesis in triploid apomicts of homosporous ferns [38,39]. The variation in spore size within and between these taxa is reflected by the inability to determine ploidy level (beyond the difference between diploid and polyploids) from spore size measurements in this complex [29].

Overall, our findings challenge the utility of spore size for inferring ploidy level within ferns. Nevertheless, further investigations are needed to test our findings for spore size in a greater number of lineages, and also determine the effect, if any, of reproductive mode on the relationship between DNA amount and spore size.

### The evolution of genome size in the *Asplenium monanthes* complex

The high chromosome numbers, and conserved chromosome sizes reported for many homosporous ferns has contributed to the hypothesis that the evolution of fern genomes is less dynamic than the evolution of angiosperm genomes [3,4,9]. This has been suggested to be due to a higher retention rate of chromosomes and the possible suppression of transposable elements (TEs) in homosporous ferns [5,6]. The inferred constancy of chromosome size is based on physical measurements [16], the low number of reported retrotransposons [17], and the reported correlation between chromosome number and genome size [6]. Bainard *et al*. [6] suggest that these observations show that genome size expansion is mainly driven by polyploidy, and that stepwise increases observed in 1C*x*-values across monilophytes might be indicative of paleopolyploidy.

Indeed, in the *Asplenium monanthes* complex studied here some of the 1C-value variation within clades clearly arises from polyploidisation, with ploidy levels ranging from 2*x* to 4*x* (Table 4), as inferred by comparing the genome size data with previously published chromosome counts (available for some of the species). This is consistent with the hypothesis that genomes size variation in homosporous ferns is driven by polyploidisation. However, contrary to this hypothesis, we also report variation in the monoploid genome size (1C*x*-value), indicating chromosome size variation between species (Figure 3). Our study provides evidence that both processes of genome evolution are occurring in this complex, both are discussed below.

Polyploidy is known to occur in several species of this complex based on previously reported chromosome counts (see Methods). The genome size data obtained in this study proved useful in identifying putative cryptic species (e.g. *A*.aff.*heterochroum*, and MO2,II) and in inferring ploidy levels for taxa without reported chromosome numbers. Inferences of ploidy were carried out with caution as even closely related taxa with different ploidy levels can display similar genome sizes, potentially leading to incorrect ploidy determinations [40]. *A.formosum* and the apomict *A.resiliens* clearly illustrated this situation as both taxa have very similar 2C-values (mean 2C = 13.61 pg and 13.99 pg respectively) (Figures 1 and 3). Without additional information it might be tempting to assume they had the same ploidy level. Yet complementary information from published chromosome counts strongly supported the presence of different ploidy levels, with *A.formosum* being a sexual diploid and *A.resiliens* a triploid apomict. Different ploidy levels were also identified among *A*.aff.*heterochroum* specimens (RD75 = 3*x* and RD9a = 4*x*), when compared to the genome size of the sister species *A.resiliens*.

In the *A.monanthes* complex moderate variation in 1C-values was noted between the accessions identified as *A.monanthes* (Figure 1). However, in all but one case (i.e. RD96), these specimens were inferred to be triploid. It is suggested that such variation has most likely arisen from differences in the DNA contents of the progenitor species that gave rise to the triploids, a claim supported by the different 1C-values reported for the putative diploid progenitor species, spec.nov.1 and spec.nov.2 [29]. The exception noted in specimen RD96, which belonged to the MO2.II lineage of *A.monanthes*, was inferred to be a diploid apomict, due to a significantly smaller 2C-value (18.58 pg) than the mean 2C of the remaining triploid taxa (27.64 pg). This suggestion is consistent with the occurrence of diploid apomicts in other ferns, including taxa of the *Dryopteris affinis* complex [19,41] and the *Pteris cretica* complex [42].

Chromosome size expansion/reduction is indicated in this complex by the observed differences in the monoploid genome size of the study species. This finding would suggest that chromosome size may not be as conserved as widely reported in homosporous ferns (e.g. [3]). In our study, the 1C*x*-values of specimens within the *A.resiliens* clade (1C*x* = 4.66-4.79 pg) are similar to more distantly related *Asplenium* species including *A.trichomanes* ssp. *quadrivalens* (1C*x* = 4.53 pg; a species within *A.trichomanes* complex) [6,8]. This indicates that *A.formosum* and species within the *A.monanthes* clade have undergone up to a two-fold expansion in monoploid genome size.

An expansion of the chromosome/ monoploid genome size could arise via retrotransposon-driven changes, as suggested for *Equisetum* and *Pstilotum*[9,38,43,44]. Retrotransposon proliferation and elimination is linked with genomic and environmental factors such as effective population size, environmental stress, hybridisation and polyploidy [13,45-49]. Given that the apomictic *A.monanthes* clade and related lineages show strong patterns of reticulate evolution, it is possible that these processes may be acting as triggers for retrotransposon activity leading to the range of 1C*x*-values observed. Indeed, given the extent of hybridization and reticulate evolution reported in homosporous ferns in general [50], it seems likely that retrotransposon driven changes in genome size are probably more widespread across ferns but may have been largely overlooked due to the low level of sampling.

## Conclusions

Our findings indicate that the evolution of genome size and spore size are not correlated within the *A.monanthes* complex. These findings challenge the utility of spore size for inferences of ploidy level within ferns. However, the prevalence of apomixis within this complex may be the cause of these findings, and the effect, if any, of reproductive mode on the relationship between DNA amount and spore size is currently unclear.

Our study also provides important insight into the dynamism of the fern genome. Previous studies have suggested that chromosome size expansion plays only a minor role in the evolution of the fern genome; with most genome size variation generated by polyploidisation. These findings were based on a broad taxonomic sample but included a very low coverage (<1%) of homosporous fern species. In our analysis of genome size in a polyploid species complex, we have found evidence to suggest that genome size variation is not explained by polyploidy alone, but also by mechanisms inducing changes in the amount of DNA per chromosome (chromosome size), without altering the number of chromosomes per genome. This finding indicates the potential for retrotransposon-driven chromosome/genome size expansion within homosporous ferns. This would have large implications for our understanding of the evolution of homosporous fern genomes in general, and it highlights the need for a substantial increase in genome size sampling, in order to determine the full extent to which these processes operate across the diversity of ferns.

## Methods

### Taxa studied

The *Asplenium* species included in the present study are listed in Table 2. They were selected based on the phylogenetic investigation of the complex by Dyer *et al.*[29] and whether the species could be successfully cultivated (see Table 2). Spec.nov1 and spec.nov.2, which belong to the *A.monanthes* complex, are reported by Dyer *et al*. [29] to be sexual diploids (based on spore size and nuclear DNA sequence analysis) and considered to be putative progenitor species to the two distinct apomictic lineages of *A.monanthes*, referred to as MO1 and MO2 (see Figure 1).

Chromosome counts and derived ploidy levels are reported in the literature for: *A.formosum,* 2*n* = 2*x* = 72 [1,51-54]; *A.monanthes*, mainly 2*n* = 3*x* = 108, although there is a single count of 2*n* = 4*x* = 144 [38,55-60]; *A.resiliens*, 2*n* = 3*x* = 108 and *A.heterochroum*, 2*n* = 4*x* = 144/2*n* = 6*x* = 216, 2*n* = 5*x* = 180 [52,57,61-67].

Fresh material was unavailable for *A.fibrillosum, A.polyphyllum, A.soleirolioides*, spec.nov.1, spec.nov.2, and some accessions of *A.formosum* (see Table 2), so silica dried samples were used instead. In total, the study sample comprised 31 fresh samples cultivated from specimen spores, and seven silica-dried samples.

### Flow cytometry: genome size and DNA ploidy

The nuclear DNA content of 38 specimens was measured following the one-step procedure described by Dolezel *et al.*[33]. Individual sporophytes were prepared as follows: several pinnae (after removing the rachis) were co-chopped using new razor blades, together with the appropriate calibration standard (*Pisum sativum* ‘Ctirad’, 2C = 9.09 pg) [68], in a Petri dish containing 2 mL of ‘General purpose buffer’ (GPB) (Loureiro *et al*., [69]) supplemented with of 3% PVP-40 . The suspension of nuclei was then filtered through a 30 μm nylon mesh, stained with 100 μl of propidium iodide (Sigma; 1 mg · mL^-1^), and treated with 34 μl of 3 mg · mL^-1^ ribonuclease A (RNase A; Sigma). Both propidium iodide and RNase A were adjusted to a final concentration of 50 μg/mL. Samples were kept on ice for 30 min and 5,000 particles recorded using a Partec Cyflow SL3 flow cytometer (Partec GmbH) fitted with a 100 mW green solid state laser (532 nm, Cobolt Samba). Flow histograms were analysed with the FlowMax software (v. 2.4, Partec GmbH). Three sporophytes were measured separately for each specimen, and three replicates of each were processed. Measurements obtained from fresh and silica dried materials of the same specimens were compared to determine the extent to which the preservation method influenced the relative fluorescence (i.e. nuclear DNA content) estimate.

We compared 1C*x*-values within species (for species with karyologically determined ploidy levels) to check for cryptic ploidy levels. In order to infer DNA ploidy levels (i.e. ploidy levels inferred only from DNA amount cf. [40]) for the species without chromosome counts, we compared their holoploid genome size and mean spore length (see section below), with those of taxa within the same clades whose ploidy level had been karyologically determined. Given the comprehensive variety (quantity and geographic spread) of the previously reported karyological data, we consider our inferences of 1C*x*-values for the remaining taxa (by means of the DNA content) to be legitimate.

### Spore measurements

To analyse the relationship between spore size and genome size we combined the spore size data from Dyer *et al*. [29], with additional spore measurements made for the remaining specimens sampled in this study. Spores from individual specimens were mounted onto slides using glycerine jelly. Each spore length was measured using AxioVision on a calibrated light-microscope (v4.8.2, Zeiss). An average of 25 spores were measured per specimen, and a mean spore length was calculated (Table 2). Special care was taken to identify putative abortive spores that were then excluded from the analyses. Individual box plots were compiled to show spore size variance within each specimen, as well as the interquartile range and the median.

### The relationship between DNA amount and spore length

We investigated the relationship between DNA amount and spore length by comparing the results obtained from analysing the raw data with those obtained using phylogenetic independent contrasts (PIC),. A standard regression analysis assumes that the data points are independent, which is not the case in related species data. PIC’s take phylogenetic relationships into account by transforming the species data into statistically independent data. Different DNA amount values were used for specimens based on their mode of reproduction: apomictic ferns produce unreduced spores, and so 2C-values were used for analysis; sexually reproducing ferns produce reduced spores, and hence 1C-values were used for analysis. The raw data were not normally distributed; therefore, in order to linearize the data for PIC analysis [70], the mean measurements for DNA amount were log transformed.

#### Regression analysis of raw data

We used a linear model (LM) regression analysis to test for the correlation between traits in all sampled specimens. We then fitted a line of best fit using a standardised major axis (SMA) to obtain a slope estimate and *r*^*2*^ value (Beaulieu *et al*., [24]; Connolly *et al*., [71]). The SMA reduces the residuals in both the dependent and independent variables (rather than just the dependent variable, as in the LM model), and is therefore useful here as it is unknown which variable is which. An SMA was fitted using the (S)MATR package in R [72,73].

#### Regression analysis incorporating phylogenetic data

To incorporate phylogenetic information into the regression analysis using PIC, we first reconstructed a phylogenetic tree of the sampled taxa. Bayesian inference (BY) was performed on a combined matrix of three plastid regions for the species listed in Table 2 (see [29], for voucher and accession numbers), using substitution models determined in jModeltest according to BIC criterion [74]. Sequence data were incomplete for some of the taxa sampled in this study (i.e. not all three plastid regions were present). On these occasions, sequence data from very closely related taxa (based on analysis of individual plastid regions) were used as substitutes in phylogenetic reconstruction (see Table 2). Analysis was carried out in MrBayes 3.1 [75,76], with Markov Chain Monte Carlo (MCMC) run for 5 million generations and sampled every 500 generations to approximate the posterior probabilities of trees. Two analyses were run simultaneously, and a conservative burn-in phase of 25% was implemented to disregard trees prior to convergence on the maximum likelihood. Remaining trees were then compiled to give 7,500 trees for each run, from which a 50% majority rule consensus was calculated.

#### Phylogenetically independent contrasts (PIC)

In order to determine whether PIC analysis was appropriate, we tested for ‘phylogenetic signal’ (= κ value), i.e., trait similarity among closely related species [77]. We used the ‘Analysis of Traits’ model in Phylocom [78], using the ‘Picante package’ in R [79], to asses phylogenetic signal for a series of different branch length transformations. Significant phylogenetic signal was shown for all branch length transformation (Table 3), supporting phylogenetic regression by the PIC method [37,80].

PICs were calculated using the PDAP: PDTREE module in Mesquite v.2.75 [81,82]. This method uses branch lengths to standardise contrasts between closely related taxa and is able to deal with the soft polytomies present in our phylogeny (Garland and Díaz-Uriarte [83]). We had several zero-length terminal branches, so it was necessary to transform branch lengths in Mesquite, in order to generate the PICs.

To test whether branch lengths had adequately standardised the contrasts, we performed regression analysis on the PICs of both characters against their standard deviation. Logarithmic, Pagel [84] and Nee [85] transformation of branch lengths resulted in an insignificant relationship for spore length, but was significant for DNA content (see Table 3) indicating that the contrasts had not been significantly standardised. However, the branch transformation methods of Grafen [86], showed no significant relationship between contrasts and their standard deviations for both characters (DNA content, p = 0.289; Spore length, p = 0.090) (see Table 3), indicating the contrasts had been adequately standardised by branch lengths. The contrasts were then standardised by dividing them by their respective standard deviations. The sign of the DNA amount contrasts were made positive, and the spore length contrasts were compared in the same direction across the node [37]. Regression analysis of standardised contrasts (forced through the origin) was performed to test for the correlated evolution of traits in R [37,81]. The slope estimate and *r*^*2*^ value was obtained using SMA analysis in R (as above), and forced through the origin.

## Abbreviations

TE: Transposable element; CV: Coefficient of variation; CI: Confidence interval; PIC: Phylogenetically independent contrasts; LM: Linear model; SMA: Standardised major axis; (S)MATR: Standardised major axis tests and routines; BY: Bayesian; BIC: Bayesian information criterion; MCMC: Markov Chain Monte Carlo.

## Competing interests

The authors declare that there are no competing interests concerning the content and publication of this manuscript.

## Authors’ contributions

RJD designed the study, carried out data collection and statistical analyses, and drafted the manuscript. JP participated in the design of the study, and helped with the acquisition of genome size data and the drafting of the manuscript. IJL participated in the design of the study, and helped with statistical analyses and the drafting of the manuscript. VS and HS participated in the conception of the study and drafting of the manuscript. All authors have read and approved the final manuscript.

## Authors’ information

Ilia J Leitch and Harald Schneider are joint senior authorship.
